# Hybrid time series models with exogenous variable for improved yield forecasting of major *Rabi* crops in India

**DOI:** 10.1038/s41598-023-49544-w

**Published:** 2023-12-14

**Authors:** Pramit Pandit, Atish Sagar, Bikramjeet Ghose, Prithwiraj Dey, Moumita Paul, Saeed Alqadhi, Javed Mallick, Hussein Almohamad, Hazem Ghassan Abdo

**Affiliations:** 1 Department of Agricultural Statistics & Computer Application, Rabindra Nath Tagore Agriculture College, Birsa Agricultural University, Ranchi, 834006 India; 2 Department of Agricultural Engineering, Rabindra Nath Tagore Agriculture College, Birsa Agricultural University, Ranchi, 834006 India; 3 Department of Agricultural Statistics, Bidhan Chandra Krishi Viswavidyalaya, Mohanpur, 741252 India; 4 Agricultural and Food Engineering Department, Indian Institute of Technology Kharagpur, Kharagpur, 721302 India; 5 Department of Civil Engineering, College of Engineering, King Khalid University, Abha, Kingdom of Saudi Arabia; 6 Department of Geography, College of Arabic Language and Social Studies, Qassim University, Buraydah, 51452 Saudi Arabia; 7 Geography Department, Faculty of Arts and Humanities, Tartous University, Tartous, Syria

**Keywords:** Ecology, Evolution, Environmental sciences

## Abstract

Accurate and in-time prediction of crop yield plays a crucial role in the planning, management, and decision-making processes within the agricultural sector. In this investigation, utilizing area under irrigation (%) as an exogenous variable, we have made an exertion to assess the suitability of different hybrid models such as ARIMAX (Autoregressive Integrated Moving Average with eXogenous Regressor)–TDNN (Time-Delay Neural Network), ARIMAX–NLSVR (Non-Linear Support Vector Regression), ARIMAX–WNN (Wavelet Neural Network), ARIMAX–CNN (Convolutional Neural Network), ARIMAX–RNN (Recurrent Neural Network) and ARIMAX–LSTM (Long Short Term Memory) as compared to their individual counterparts for yield forecasting of major *Rabi* crops in India. The accuracy of the ARIMA model has also been considered as a benchmark. Empirical outcomes reveal that the ARIMAX–LSTM hybrid modeling combination outperforms all other time series models in terms of root mean square error (RMSE) and mean absolute percentage error (MAPE) values. For these models, an average improvement of RMSE and MAPE values has been observed to be 10.41% and 12.28%, respectively over all other competing models and 15.83% and 18.42%, respectively over the benchmark ARIMA model. The incorporation of the area under irrigation (%) as an exogenous variable in the ARIMAX framework and the inbuilt capability of the LSTM model to process complex non-linear patterns have been observed to significantly enhance the accuracy of forecasting. The performance supremacy of other hybrid models over their individual counterparts has also been evident. The results also suggest avoiding any performance generalization of individual models for their hybrid structures.

## Introduction

Agriculture plays a crucial role in the economy and sustenance of societies across India. The reliance on an enormous number of farmers, intermediaries, private enterprises, and public sectors in agriculture makes this sector an integral component of the country’s development^1^. Farmers perceive agriculture as financially worthwhile only when they obtain a successful crop season that leads to abundant harvests, leading to favorable prices^2^. Therefore, anticipating crop yield is critical in the agricultural sector for planning, management, and decision-making processes. It enables planners and stakeholders to take proactive measures to ensure adequate food supply and distribution, thereby enhancing food security at local, regional, and national levels. With reliable and in-time forecasts, it is within the capabilities of governments to arrive at well-informed decisions about imports, exports, and food aid programs.

Among earlier attempts of yield prediction, crop weather models developed by Fisher^3^ and Baier^4^ are noteworthy. Of late, remote sensing (RS)-based approaches^5,6^ and different simulation techniques^7,8^ have gained traction due to the considerable benefits in tracking farm yield and operations over the cultivation period. Models for yield prediction based on the physiological characteristics of crops are also in vogue^9,10^. However, these approaches may not be suitable at the macro level due to economic and data availability constraints, whereas thanks to their relative ease of use, statistical models can be effectively deployed for forecasting tasks.

The Autoregressive Integrated Moving Average (ARIMA) model stands as a crucial and frequently utilized model for time series analysis. The ARIMAX (ARIMA with eXogenous Regressor) model, an enhanced version of the ARIMA model, has been leveraged as well for quantitative understanding of crop responses. It offers flexibility by including pertinent auxiliary variable(s) through a linear modeling structure. However, both ARIMA and ARIMAX models suffer from the presumption of linearity. Over the past thirty years, a significant amount of literature has emerged that focuses on the modeling of non-linear characteristics in time series data.

The escalating studies in numerous machine learning (ML) algorithms, accompanied by their plenty of successful forecasting applications^11–14^, position them as viable candidates for time series forecasting. Contrary to the conventional models, these ML approaches are data-driven, self-adaptive, non-linear, and non-parametric with few restrictive presumptions. Nevertheless, given the inherent mixture of linear and non-linear patterns observed in agricultural data, it is impractical to rely solely on a single linear or non-linear model to effectively capture all the features exhibited by these time series data. In such cases, hybrid modeling strategies, i.e., sequential implementation of linear and non-linear models, have consistently shown better outcomes^15–19^.

Several authors have put efforts into comparing traditional, ML, and hybrid methodologies in their studies. Kumar et al.^20^ compared ARIMA and Time-Delay Neural Network (TDNN) models for potato price forecasting in India. In their analysis, the TDNN model handled non-stationary, non-linear, and non-normal aspects of the datasets concurrently, outperforming the classical ARIMA model. Rathod et al.^21^ predicted banana production in Karnataka by employing hybrid models. These models integrated the ARIMA model with the TDNN and Non-Linear Support Vector Regression (NLSVR) models, respectively. The hybrid models exhibited superior forecasting accuracies in comparison to the individual models, as evidenced by empirical results. The findings of Rathod and Mishra^22^ were also in consonance, whereby similar hybrid combinations were compared to the individual and stepwise regression models. Supriya^23^ discovered that the sequential combination of the ARIMAX–Artificial Neural Network (ANN) model outperformed the component models in predicting the damage caused by the yellow stem borer in the Telangana State of India. Neog et al.^18^ used ARIMAX–ANN and ARIMAX–SVM hybrid combinations for forecasting autumn rice production to demonstrate their competitive advantages over the corresponding single linear and non-linear models.

The aforementioned facts clearly demonstrate that the hybrid models employed so far mostly consist of either TDNN or NLSVR for modeling the non-linear residuals. As many advanced neural network models, such as Wavelet Neural Network, Convolutional Neural Network, Recurrent Neural Network, Long Short Term Memory network, etc., have recently been included in the model builders’ arsenal, their performance needs to be examined in the hybrid structure. The literature review further reveals a scarce amount of research in the realm of agricultural yield forecasting, especially using hybrid techniques with exogenous variables.

Among the different factors of production, irrigation plays a crucial role in enhancing crop yields and ensuring food security on a global scale^24,25^. Countries like the United States, China, Russia, and Australia have made significant investments in irrigation infrastructure, establishing themselves as major wheat producers^26–28^. In the context of wheat production in India, irrigation has transformed agriculture notably in the ‘wheat belt’ states of Punjab, Haryana, and Uttar Pradesh^29–34^. In the case of sugarcane, which is also a water-intensive crop, consistent supply of water via irrigation networks, such as canals, wells, and sprinklers ensures the necessary moisture for sugarcane growth^35–38^. Similarly, the adoption of irrigation techniques has resulted in a substantial increase in groundnut yields in India^39,40^.

With this backdrop, this study focuses on forecasting the yield of major *Rabi* crops in India, particularly by utilizing area under irrigation (%) as an exogenous variable. The key content of the paper includes a comparative assessment of different ARIMAX-based hybrid models and their individual counterparts within the purview of forecasting major *Rabi* crop yields in India. This study incorporates a consideration of the ARIMA model’s performance as a benchmark. Yield forecasts for the next five years (2021–2025) have also been obtained by the best-performing model to facilitate policymakers and stakeholders in their decision-making processes.

## Materials and methods

### Data

Yearly data on yield (Kg./Hectare) and area under irrigation (%) of India for wheat, sugarcane, and groundnut have been collected and compiled from the various issues of ‘Agricultural Statistics at a Glance’ published by Economics & Statistics Division, Department of Agriculture and Farmers Welfare, Ministry of Agriculture and Farmers Welfare, Government of India (www.agricoop.nic.in and http://desagri.gov.in). The required time series data are available for the period of 1950 to 2019 for wheat and sugarcane and from 1952 to 2019 in the case of groundnut. Methodologically, for each model, the last seven observations have been retained for testing purpose and the remaining observations have been utilized for model building.

### Time series models

#### Autoregressive integrated moving average (ARIMA) model

The most commonly used models to represent linear dynamics in time series literature are the ARIMA models^41^. We state that a univariate process $\{{{\text{y}}}_{{\text{t}}}\}$ conforms to the ARIMA (p, d, q) model if it can be expressed as follows:

1 $$\phi_{{\text{p}}} \left( {\text{B}} \right)\nabla^{{\text{d}}} {\text{y}}_{{\text{t}}} = {\uptheta }_{{\text{q}}} \left( {\text{B}} \right){\upvarepsilon }_{{\text{t}}}$$

where p, d, and q represent the orders of autoregression, differencing, and moving average, respectively.

2 $$\phi_{{\text{p}}} \left( {\text{B}} \right) = 1 - \sum\nolimits_{{{\text{i}} = 1}}^{{\text{p}}} {\phi_{{\text{i}}} {\text{B}}^{{\text{i}}} = 1 - \phi_{1} {\text{B}} - \phi_{2} {\text{B}}^{2} - \cdots - \phi_{{\text{p}}} {\text{B}}^{{\text{p}}} }$$

3 $${\uptheta }_{{\text{q}}} \left( {\text{B}} \right) = 1 - \sum\nolimits_{{{\text{j}} = 1}}^{{\text{q}}} {{\uptheta }_{{\text{j}}} {\text{B}}^{{\text{j}}} = 1 - {\uptheta }_{1} {\text{B}} - {\uptheta }_{2} {\text{B}}^{2} - \cdots - {\uptheta }_{{\text{q}}} {\text{B}}^{{\text{q}}} }$$

4 $$\nabla^{{\text{d}}} = \left( {1 - {\text{B}}} \right)^{{\text{d}}} \,{\text{and}}\,{\text{B}}\left( {{\text{y}}_{{\text{t}}} } \right) = {\text{y}}_{{{\text{t}} - 1}}$$

{${\upvarepsilon }_{{\text{t}}}$} is hypothesised to adhere to a standard white noise process, exhibiting a normal distribution with zero mean and a variance of ${\upsigma }^{2}$. In case ${{\text{y}}}_{{\text{t}}}$ does not undergo mean-adjustment, there is provision to append a constant term, denoted by μ, to the right side of Eq. (1). The ARIMA methodology can be segmented into three essential stages: identification, estimation, and diagnostic checking. In the identification stage, the parameters for the ARIMA model are provisionally chosen. These tentatively selected parameters are then quantified in the estimation stage. During the subsequent diagnostic checking stage, the model adequacy is evaluated thoroughly. If the model is deemed unsuitable, the entire three-step process resumes and continues until an apt ARIMA model for the given time series is obtained.

#### Autoregressive integrated moving average with exogenous regressor (ARIMAX) model

The ARIMAX model is a more sophisticated version of the ARIMA model. It has the ability to incorporate an external input variable^42^. It operates by assuming a form predicated on a given historical input vector ${{\text{x}}}_{{\text{t}}}$:

5 $$\phi_{{\text{p}}} \left( {\text{B}} \right)\nabla^{{\text{d}}} {\text{y}}_{{\text{t}}} = {\upmu } + {\upnu }\left( {\text{B}} \right){\text{x}}_{{{\text{mt}}}} + {\uptheta }_{{\text{q}}} \left( {\text{B}} \right){\upvarepsilon }_{{\text{t}}}$$

where $\phi_{{\text{p}}} \left( {\text{B}} \right)$ and ${\uptheta }_{{\text{q}}} \left( {\text{B}} \right)$ assume the predefined form of the ARIMA model and ${\upnu }\left( {\text{B}} \right){\text{x}}_{{{\text{mt}}}}$ is defined as:

6 $${\upnu }\left( {\text{B}} \right){\text{x}}_{{{\text{mt}}}} = {\upbeta }_{1} {\text{x}}_{{1{\text{t}}}} + {\upbeta }_{2} {\text{x}}_{{2{\text{t}}}} + \cdots + {\upbeta }_{{\text{m}}} {\text{x}}_{{{\text{mt}}}}$$

In the model, m denotes the number of exogenous input variables. Additionally, it is presumed that $\{{\upvarepsilon }_{{\text{t}}}\}$ follows a white noise process with ${\text{N}}(0, {\upsigma }^{2})$.

#### Time-delay neural network (TDNN) model

Artificial neural networks, modelled after the human brain, are composed of mathematical functions known as artificial neurons (nodes). These neurons are grouped together to form a layer of processing elements. Typically, neural networks are structured with three layers: the input, hidden, and output layers.

An approach to forecast time series with neural networks involves incorporating dynamic characteristics into a static structure, like a multilayer perceptron. This approach offers an implicit functional depiction of time, which is useful in portraying the behavior of data that evolves over time^43^. A potential approach to incorporate short-term memory is using time delay as input^44^. TDNN exemplifies such a structure. The general expression for the final output ${{\text{y}}}_{{\text{t}}}$ of a TDNN model is expressed as follows:

7 $${\text{y}}_{{\text{t}}} = {\text{f}}\left( {{\upalpha }_{0} + \sum\nolimits_{{{\text{j}} = 1}}^{{\text{q}}} {{\upalpha }_{{\text{j}}} {\text{g}}\left( {{\upbeta }_{{0{\text{j}}}} + \sum\nolimits_{{{\text{i}} = 1}}^{{\text{p}}} {{\upbeta }_{{{\text{ij}}}} {\text{y}}_{{{\text{t}} - {\text{i}}}} } } \right)} } \right) + {\upvarepsilon }_{{\text{t}}}$$

where ${\upalpha }_{{\text{j}}}$ and ${\upbeta }_{{{\text{ij}}}}$ are the model hyper-parameters. p and q denote the number of input and hidden nodes, respectively. The hidden layer activation function (g) has taken the form of Rectified Linear Unit (ReLU).

8 $${\text{f}}\left( {\text{y}} \right) = {\text{y}}^{ + } = {\text{maximum}}\left( {0,{\text{y}}} \right)$$

The identity function operates as the output layer activation function (f).

#### Non-linear support vector regression (NLSVR) model

NLSVR primarily converts the initial input space into a feature space with higher dimensions. Subsequently, a linear regression model is built within it, effectively representing non-linear regression in the original space^21,45^. In the context of a dataset represented by ${\text{Z}}={\{{{\text{x}}}_{\mathrm{i }}{{\text{y}}}_{{\text{i}}}\}}_{{\text{i}}=1}^{{\text{N}}}$, where the input vector ${{\text{x}}}_{{\text{i}}}$ belongs to the n-dimensional real space, ${{\text{y}}}_{{\text{i}}}$ represents the scalar output, and N denotes the size of Z, the general equation of NLSVR can be expressed as follows:

9 $${\text{f}}\left( {\text{x}} \right) = {\text{w}}^{{\text{T}}} \phi \left( {\text{x}} \right) + {\text{b}}$$

where w represents the weight vector, $\phi \left( {\text{x}} \right)$ stands for the non-linear mapping function, b signifies the bias term, and superscript T indicates the transpose operation. The data is used to estimate the coefficients w and b through the minimization of a regularized risk function:

10 $${\text{R}}\left( {\uptheta } \right) = \frac{1}{2}\|{\text{w}}\|^{2} + {\text{C}}\left[ {\frac{1}{{\text{N}}}\sum\nolimits_{{{\text{i}} = 1}}^{{\text{N}}} {{\text{L}}_{{\upvarepsilon }} \left( {{\text{y}}_{{\text{i}}} ,{\text{ f}}\left( {{\text{x}}_{{\text{i}}} } \right)} \right)} } \right]$$

Equation (10) comprises two elements: the first is a regularized component represented as half times the norm of ${\text{w}}$ squared, while the second component is referred to as the empirical error, denoted as $\frac{1}{{\text{N}}}\sum\nolimits_{{{\text{i}} = 1}}^{{\text{N}}} {{\text{L}}_{{\upvarepsilon }} \left( {{\text{y}}_{{\text{i}}} ,{\text{ f}}\left( {{\text{x}}_{{\text{i}}} } \right)} \right)}$. The regularized risk function effectively balances the optimization of both of these components simultaneously, thus preventing both underfitting and overfitting issues of the model.

#### Wavelet neural network (WNN) model

In a neural network configuration, different wavelet functions can be effective for approximating a function or predicting output data. Because of this, wavelet functions can serve as activation functions within hidden neurons^46^. This concept leads to the formulation of the WNN. In a WNN, the activation functions are derived from an orthonormal wavelet basis. The term ‘wavelon’ is used to refer to the neurons in this context. The output of a wavelon with a single input is defined as follows:

11 $${\Psi }_{{{\upalpha },{\text{t}}}} \left( {\text{y}} \right) = {\Psi }\left( {\frac{{{\text{y}} - {\text{t}}}}{{\upalpha }}} \right)$$

Morlet function^47^ has been proposed for WNN in this study, which is expressed as follows:

12 $${\Psi }\left( {\text{y}} \right) = \exp \left( { - {\text{y}}^{2} } \right){\text{cos}}\left( {5{\text{y}}} \right)$$

This wavelet is derived from a function that bears proportionality to both the cosine function, and normal probability density function.

#### Convolutional neural network (CNN) model

CNN is a network model introduced by Lecun et al.^48^, which has a neural connectivity pattern similar to the visual cortex of animals. CNN has found extensive usage in the realm of image and natural language processing^49^. Nonetheless, it can be effectively employed for time series forecasting. One of the key advantages of CNNs lies in their ability to perceive data locally and share weights, which can greatly lessen the number of parameters and thereby improve learning efficiency. CNN consists of two structural layers: the convolutional layer and the pooling layer^50^. Within the convolution layer, there are several convolutional kernels. The process of convolution can be represented as follows:

13 $${\text{l}}_{{\text{t}}} = {\text{tanh}}\left( {{\text{x}}_{{\text{t}}} {\text{k}}_{{\text{t}}} + {\text{b}}_{{\text{t}}} } \right)$$

where tanh is the activation function. ${{\text{l}}}_{{\text{t}}}$, ${{\text{x}}}_{{\text{t}}}$, ${{\text{k}}}_{{\text{t}},}$ and ${{\text{b}}}_{{\text{t}}}$ represent the output value after convolution, the input vector, the weight of the convolution kernel, and the bias of the convolution kernel, respectively. Following the convolution operation, the main characteristics of the data are retrieved, which is marked by an expansion in the feature dimensions. To address this challenge as well as to lessen the load during training, a pooling layer is introduced before providing the final output^51^.

#### Recurrent neural network (RNN) model

RNNs pose greater technical complexity compared to feedforward networks, necessitating a solid grasp of dynamic recurrence mechanisms. A basic RNN can be thought of as a single-layer RNN where the activation is delayed and simultaneously looped back with the external input (or the output from a preceding layer). The conventional RNN can be described mathematically as^52^:

14 $${\text{h}}_{{\text{t}}} = {\upsigma }_{{\text{t}}} \left( {{\text{Uh}}_{{{\text{t}} - 1}} + {\text{Ws}}_{{\text{t}}} + {\text{b}}} \right)$$

where t (0, 1, …, N) represents a discrete time point, N signifies the final time in a finite time period, ${{\text{s}}}_{{\text{t}}}$ refers to a vector with m dimensions representing external inputs at time t, and ${{\text{h}}}_{{\text{t}}}$ represents the n-dimensional output activation via ${\upsigma }_{{\text{t}}}$. This ${\upsigma }_{{\text{t}}}$ may vary over time and can exhibit non-linear behavior. The non-indexed parameters, to be set via training, are the $\mathrm{n }\times \mathrm{ n}$ matrix U, the $\mathrm{n }\times \mathrm{ m}$ matrix W, and the $\mathrm{n }\times 1$ vector b.

#### Long short term memory (LSTM) model

**Figure 1:**
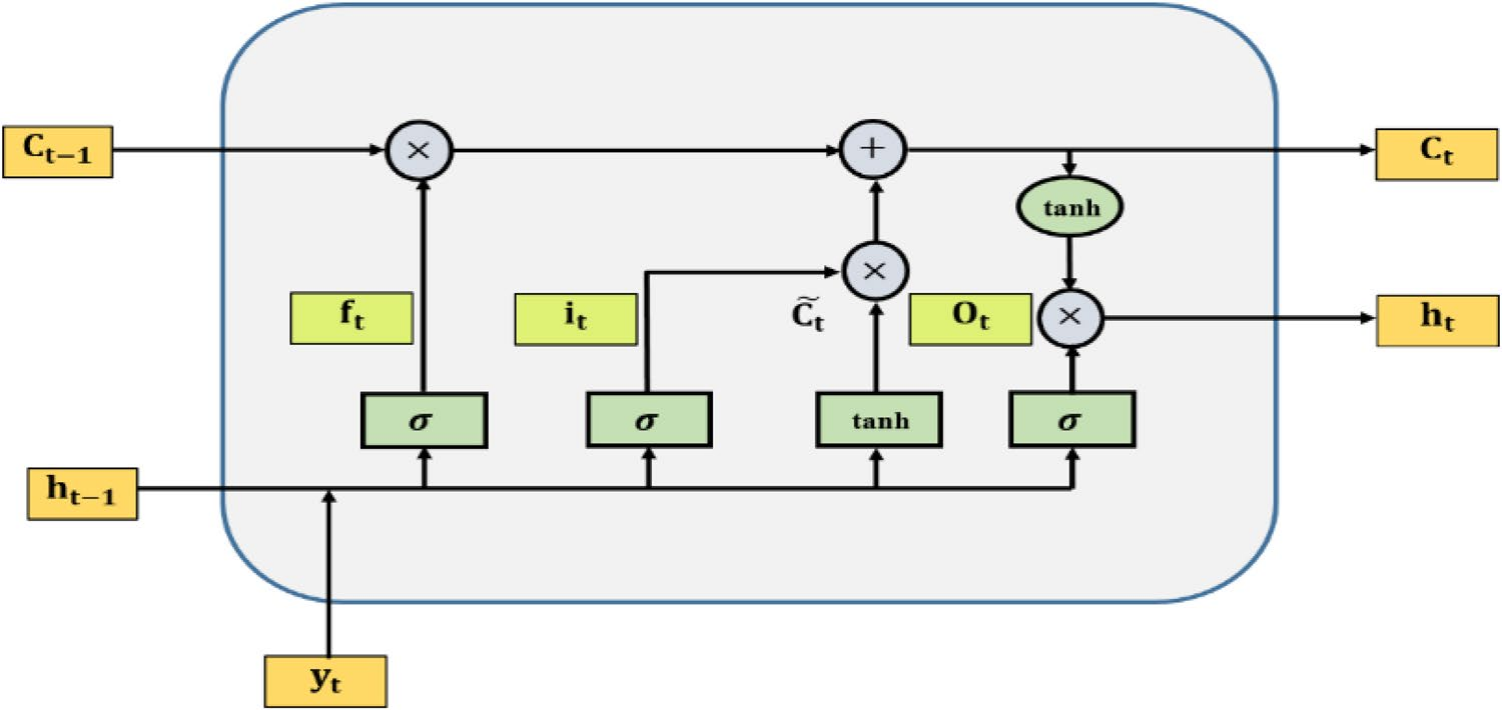
Structural representation of Long Short Term Memory.

Hochreiter and Schmidhuber^53^ proposed the LSTM neural network in 1997 to deal with long-term data dependencies. The design of LSTM allows it to recall information for an extended length of time while resolving the problem of vanishing gradient^54,55^, which is the main lacuna of the RNN model^56^. The LSTM model consists of three memory modules, namely forget gate $({{\text{f}}}_{{\text{t}}})$, input gate $({{\text{i}}}_{{\text{t}}})$, and output gate $({{\text{o}}}_{{\text{t}}})$. The primary roles of these three gates are to retain critical information while eliminating unnecessary information from the cell state. The pivotal element in LSTM is the cell state $({{\text{C}}}_{{\text{t}}})$, which operates concurrently throughout the entire recurrent chain with a few minor interactions. The structural representation of the LSTM is graphically provided in Fig. 1.

To commence the information processing with the LSTM model, the first step involves removing extraneous information from ${{\text{C}}}_{{\text{t}}}$. The forget gate offers this by employing a sigmoid function.

15 $${\text{f}}_{{\text{t}}} = {\upsigma }\left( {{\text{W}}_{{\text{f}}} \cdot \left[ {{\text{h}}_{{{\text{t}} - 1{ }}} ,{\text{ y}}_{{\text{t}}} } \right] + {\text{b}}_{{\text{f}}} } \right)$$

where ${{\text{W}}}_{{\text{f}}}$ and ${{\text{b}}}_{{\text{f}}}$ are the weight and bias of the forget gate, respectively, ${{\text{y}}}_{{\text{t}}}$ is the input value of the current time and ${{\text{h}}}_{{\text{t}}-1}$ is the output value of the prior unit.

The cell state is then updated with new or pertinent information. This is accomplished by the use of another gate with a sigmoid function, known as the input gate, and a tanh layer.

16 $${\text{i}}_{{\text{t}}} = {\upsigma }\left( {{\text{W}}_{{\text{i}}} \cdot \left[ {{\text{h}}_{{{\text{t}} - 1{ }}} ,{\text{ y}}_{{\text{t}}} } \right] + {\text{b}}_{{\text{i}}} } \right)$$

17 $$\widetilde{{{\text{C}}_{ } }}_{{\text{t}}} = {\text{tanh}}\left( {{\text{W}}_{{\text{c}}} .\left[ {{\text{h}}_{{{\text{t}} - 1{ }}} ,{\text{ y}}_{{\text{t}}} } \right] + {\text{b}}_{{\text{c}}} } \right)$$

where ${{\text{W}}}_{{\text{i}}}$, ${{\text{b}}}_{{\text{i}},}$ and ${{\text{W}}}_{{\text{c}}}$, ${{\text{b}}}_{{\text{c}}}$ are the weight and bias of the input gate and the candidate input, respectively.

In the next step, the current cell state is updated as follows:

18 $${\text{C}}_{{\text{t}}} = {\text{f}}_{{\text{t}}} \times {\text{C}}_{{{\text{t}} - 1}} + {\text{i}}_{{\text{t}}} \times \widetilde{{{\text{C}}_{ } }}_{{\text{t}}}$$

Then, the output gate takes ${\text{h}}_{{{\text{t}} - 1{ }}}$ and ${\text{y}}_{{\text{t}}}$ as input values, and its output is calculated using the formula:

19 $${\text{o}}_{{\text{t}}} = {\upsigma }\left( {{\text{W}}_{{\text{o}}} .\left[ {{\text{h}}_{{{\text{t}} - 1{ }}} ,{\text{ y}}_{{\text{t}}} } \right] + {\text{b}}_{{\text{o}}} } \right)$$

where ${{\text{W}}}_{{\text{o}}}$ and ${{\text{b}}}_{{\text{o}}}$ are the weight and bias of the output gate, respectively.

Finally, the output of the LSTM model is computed as follows:

20 $${\text{h}}_{{\text{t}}} = {\text{o}}_{{\text{t}}} \times \tanh \left( {{\text{C}}_{{\text{t}}} } \right)$$

#### Hybrid models

Hybrid time series models leverage various modeling approaches to enhance the accuracy and robustness of forecasts. These models consider the time series ${{\text{y}}}_{{\text{t}}}$ as a blend of both linear and non-linear elements.

21 $${{\text{y}}}_{{\text{t}}}={{\text{L}}}_{{\text{t}}}+{{\text{N}}}_{{\text{t}}}$$

where ${{\text{L}}}_{{\text{t}}}$ and ${{\text{N}}}_{{\text{t}}}$ represent the linear and non-linear components, respectively.

The operational premise of the hybridization approach^57^ begins with fitting a linear model to the data and obtaining the corresponding forecast $(\widehat{{{\text{L}}}_{{\text{t}}}})$. The subsequent stage entails acquiring the residuals (${{\text{e}}}_{{\text{t}}}$) of the linear model and checking for the existence of non-linear patterns in its structure.

22 $${\text{e}}_{{\text{t}}} = {\text{y}}_{{\text{t}}} - \widehat{{{\text{L}}_{{\text{t}}} }}$$

Once the residuals validate the presence of non-linearity, they are subsequently fed into an appropriate non-linear model. After obtaining forecasts $(\widehat{{{\text{N}}}_{{\text{t}}}})$ for the non-linear component, these are combined with the linear forecasts to generate the aggregate forecasts.

23 $$\widehat{{{\text{y}}_{{\text{t}}} }} = \widehat{{{\text{L}}_{{\text{t}}} }} + \widehat{{{\text{N}}_{{\text{t}}} }}$$

**Figure 2:**
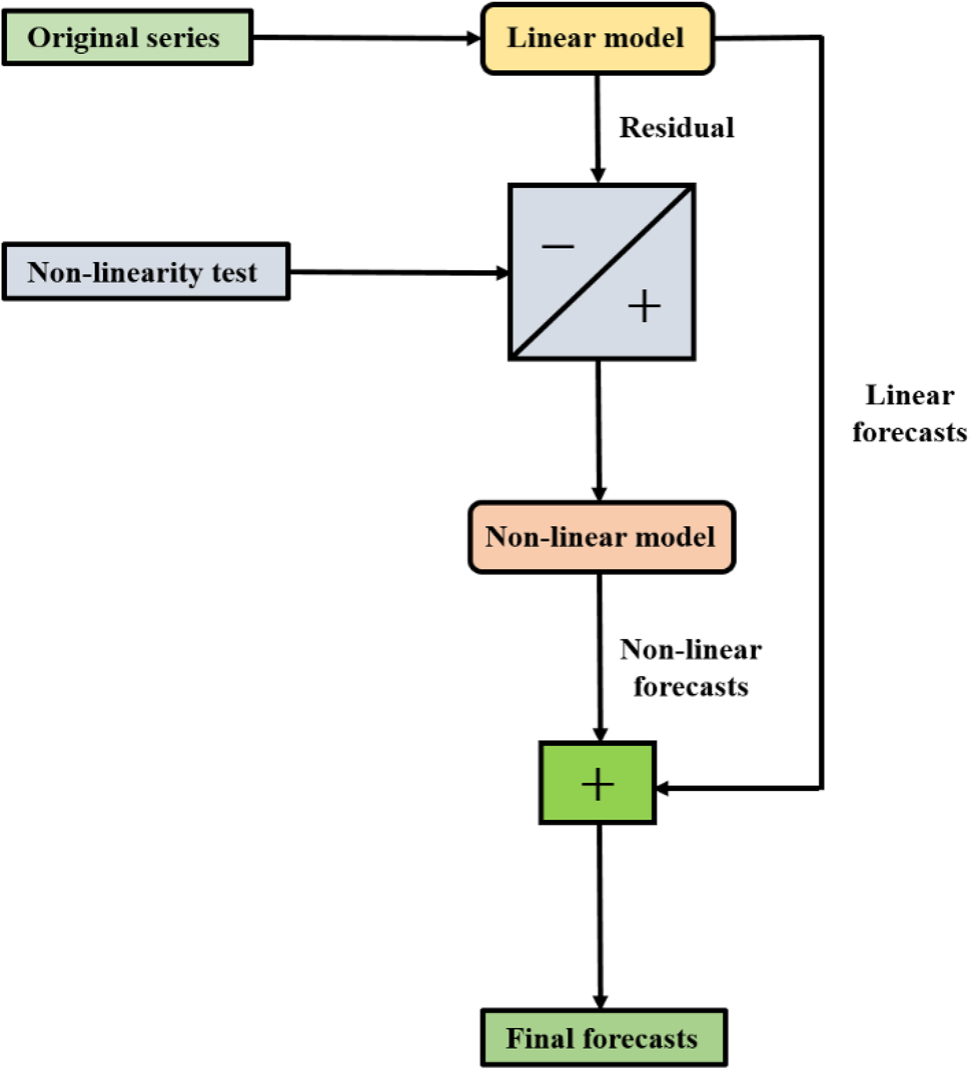
Schematic diagram of hybrid modelling strategy.

In this investigation, we have used the ARIMAX model to capture the linear component, whereby the residuals are modelled separately by the TDNN, NLSVR, WNN, CNN, RNN, and LSTM models to examine the performance of different combinations. Figure 2 provides the schematic representation of the hybridization technique.

### Assessment of forecasting accuracy

To assess the forecasting accuracy of the time series models under investigation, two performance measures, namely the root mean square error (RMSE) and the mean absolute percentage error (MAPE), are employed. The model exhibiting the lowest RMSE and MAPE values is deemed to be the most optimal.

24 $${\text{RMSE}} = \sqrt {\frac{1}{{\text{n}}}\mathop \sum \limits_{{{\text{t}} = 1}}^{{\text{n}}} \left( {{\text{y}}_{{\text{t}}} - \widehat{{{\text{y}}_{{\text{t}}} }}} \right)^{2} }$$

25 $${\text{MAPE }}\left( \%  \right) = \frac{1}{{\text{n}}}\mathop \sum \limits_{{{\text{t}} = 1}}^{{\text{n}}} \left| {\frac{{{\text{y}}_{{\text{t}}} - \widehat{{{\text{y}}_{{\text{t}}} }}}}{{{\text{y}}_{{\text{t}}} }}} \right| \times 100\%$$

where ${{\text{y}}}_{{\text{t}}}$ and $\widehat{{{\text{y}}}_{{\text{t}}}}$ denote the actual and predicted values of the t^th^ observation of the test data, respectively and n is the size of the test data set.

## Results

### Summary statistics

Table 1 displays summary statistics of the data series utilised in the investigation. Sugarcane has exhibited the highest mean irrigated area (%), with wheat and groundnut trailing behind. A high coefficient of variation value signifies considerable volatility in these series.

**Table 1:** Descriptive statistics of yield and area under irrigation (%) of major *Rabi* crops.

Statistics	Wheat		Sugarcane		Groundnut	
	Yield (Kg./Hectare)	Area under Irrigation (%)	Yield (Kg./Hectare)	Area under Irrigation (%)	Yield (Kg./Hectare)	Area under Irrigation (%)
Mean	1907.94	68.48	56,561.57	82.24	967.85	14.55
Minimum	653.00	29.37	29,495.00	64.93	554.00	1.23
Maximum	3533.00	95.76	80,497.00	96.62	2063.00	36.65
Standard Deviation	883.98	22.74	13,636.94	10.34	312.14	9.76
CV (%)	46.33	33.21	24.11	12.57	32.25	67.08
Skewness	0.05	− 0.47	− 0.34	− 0.20	1.49	0.46
Kurtosis	− 1.38	− 1.31	− 0.94	− 1.45	2.24	− 0.47

### Results of the augmented Dickey-Fuller (ADF) test

The ADF test^58,59^ has been utilised to ascertain the order of differencing and the results are presented in Table 2. All the yield series have exhibited non-stationary behavior at level series and stationary behavior at the first difference series.

**Table 2:** Results of the ADF test for testing stationarity.

Crop	Level series		First difference series	
	Statistic	*p* value	Statistic	*p* value
Wheat	− 2.85	0.23	− 4.52	< 0.01
Sugarcane	− 2.12	0.52	− 3.91	0.02
Groundnut	− 0.03	0.99	− 5.54	< 0.01

### Fitting of the ARIMA models

In the context of the ARIMA model, the autocorrelation function (ACF) and partial autocorrelation function (PACF) provide key insights into the potential order of the model. The optimal model selection is based on the minimum values for Akaike information criteria (AIC) and Bayesian information criteria (BIC), as well as the RMSE and MAPE. The parameter estimates of the chosen ARIMA models, along with their significance levels, are provided in Table 3.

**Table 3:** Parameter estimates of the fitted ARIMA models.

Crop	Model	Parameter	Estimate	*p* value
Wheat	ARIMA(2, 1, 2)	µ (Constant)	40.87	< 0.01
		${\phi }_{1}$ $({{\text{AR}}}_{1})$	− 0.18	0.38
		${\phi }_{2}$ (${{\text{AR}}}_{2})$	− 0.70	< 0.01
		${\uptheta }_{1}$ ${({\text{MA}}}_{1}$)	− 0.21	0.19
		${\uptheta }_{2}$ ${({\text{MA}}}_{2}$)	0.77	< 0.01
Sugarcane	ARIMA(3, 1, 0)	µ (Constant)	622.88	< 0.01
		${\phi }_{1}$ $({{\text{AR}}}_{1})$	− 0.31	0.01
		${\phi }_{2}$ (${{\text{AR}}}_{2})$	− 0.46	< 0.01
		${\phi }_{3}$ $({{\text{AR}}}_{3})$	− 0.26	0.03
Groundnut	ARIMA(0, 1, 1)	µ (Constant)	8.44	< 0.01
		${\uptheta }_{1}$ ${({\text{MA}}}_{1}$)	− 0.99	< 0.01

### Fitting of the ARIMAX models

The selection of a suitable exogenous variable is crucial for the ARIMAX model-building procedure. The significance of the correlation co-efficient between yield and area under irrigation (%) in each case, as reported in Table 4, indicates a possible outperformance of the ARIMAX models over the traditional ARIMA models. Following the ARIMA model-building process, the optimal model has been chosen based on the lowest AIC, BIC, RMSE, and MAPE values. Table 5 displays the specifications of the selected ARIMAX models.

**Table 4:** Correlation coefficients between yield and area under irrigation (%).

Crop	r	*p* value
Wheat	0.96	< 0.01
Sugarcane	0.96	< 0.01
Groundnut	0.83	< 0.01

**Table 5:** Parameter estimates of the fitted ARIMAX models.

Crop	Model	Parameter	Estimate	*p* value
Wheat	${{\text{ARIMAX}}(0, 1, 1)}_{\mathrm{Area under irrigation }(\mathrm{\%})}$	µ (Constant)	40.24	< 0.01
		${\theta }_{1}$ ${({\text{MA}}}_{1}$)	− 0.42	< 0.01
		${\beta }_{1}$(Regressor)	− 4.81	0.39
Sugarcane	${{\text{ARIMAX}}(0, 1, 1)}_{\mathrm{Area under irrigation }(\mathrm{\%})}$	µ (Constant)	639.05	< 0.01
		${\theta }_{1}$ ${({\text{MA}}}_{1}$)	− 0.49	< 0.01
		${\beta }_{1}$(Regressor)	306.21	0.15
Groundnut	${{\text{ARIMAX}}(0, 1, 2)}_{\mathrm{Area under irrigation }(\mathrm{\%})}$	µ (Constant)	7.62	0.02
		${\theta }_{1}$ ${({\text{MA}}}_{1}$)	− 1.22	< 0.01
		${\theta }_{2}$ ${({\text{MA}}}_{2}$)	0.40	< 0.01
		${\beta }_{1}$(Regressor)	8.97	0.07

### Results of the Broock–Dechert–Scheinkman (BDS) test

Before proceeding to non-linear or hybrid modeling strategies, it is required to examine the series for the presence of non-linear features. To assess non-linearity, the BDS test^60^ has been implemented. Table 6 provides the outcomes of the BDS test. For all three cases, a strong rejection of linearity has been observed. It implies that the non-linear models can effectively be implemented in these series.

**Table 6:** Results of the BDS test for testing non-linearity.

Crop	Epsilon	Embedding dimension = 2		Embedding dimension = 3	
		Statistic	*p* value	Statistic	*p* value
Wheat	56.43	− 1.40	0.16	− 2.62	0.01
	112.87	− 0.86	0.39	− 0.44	0.66
	169.30	− 1.23	0.22	− 1.95	0.05
	225.74	− 1.18	0.24	− 1.78	0.08
Sugarcane	1604.76	1.97	0.05	1.32	0.18
	3209.51	2.46	0.01	2.34	0.02
	4814.27	0.78	0.44	1.01	0.31
	6419.02	0.07	0.94	− 0.36	0.72
Groundnut	95.63	5.05	< 0.01	5.28	< 0.01
	191.27	3.16	< 0.01	3.65	< 0.01
	286.90	2.00	0.05	1.68	0.09
	382.53	0.70	0.48	0.51	0.61

### Fitting of the TDNN models

For this study, we have found the optimal time-delay neural network with a single hidden layer. Experimentation has been carried out to identify the number of tapped delays and hidden nodes. We have altered the range of input and hidden nodes from 1 to 6 and from 1 to 10, respectively for both the original and ARIMAX residual series. The training of networks has been accomplished by utilizing the Levenberg–Marquardt back-propagation algorithm. Table 7 contains the specifications of the chosen TDNN models.

**Table 7:** Optimal hyper-parameter values of the fitted TDNN models.

Specifications	Original series			Residual series		
	Wheat	Sugarcane	Groundnut	Wheat	Sugarcane	Groundnut
No. of input lags	3	3	4	4	1	5
No. of hidden units	6	7	4	1	2	1
Total no. of parameters	31	36	25	7	7	8

### Fitting of the NLSVR models

An essential step in NLSVR modeling is the selection of optimal hyper-parameters. The input lags, kernel function, regularization parameter, kernel width, and margin of tolerance significantly influence the NLSVR performance. In this study, we have employed the widely adopted radial basis function (RBF) as the kernel function. We have constructed NLSVR models for both the original and residual series based on the specifications outlined in Table 8.

**Table 8:** Optimal hyper-parameter values of the fitted NLSVR models.

Specifications	Original series			Residual series		
	Wheat	Sugarcane	Groundnut	Wheat	Sugarcane	Groundnut
No. of input lags	6	2	1	2	1	1
C	10.00	130.00	840.00	10.00	0.50	0.01
γ	0.25	0.25	0.80	3.00	0.25	0.25
ε	0.10	0.50	0.35	0.50	0.10	0.40

### Fitting of the WNN models

For each crop, wavelet neural network models have been chosen based on their forecasting performance at various numbers of input lags (from 1 to 6) and hidden nodes (from 1 to 10). Table 9 presents the specifications of the best performing WNN models for the original and residual series, respectively. Similar to TDNN, the Levenberg–Marquardt back-propagation algorithm has been used for training purposes.

**Table 9:** Optimal hyper-parameter values of the fitted WNN models.

Specifications	Original series			Residual series		
	Wheat	Sugarcane	Groundnut	Wheat	Sugarcane	Groundnut
No. of input lags	6	1	6	2	4	2
No. of hidden units	9	10	2	4	9	6
Total no. of parameters	73	31	17	17	55	25

### Fitting of the CNN models

The performance of the CNN model crucially relies on the optimal selection of hyper-parameters. The set of hyper-parameters tuned for the training of the CNN model consists of the number of input nodes, number of filters and kernel size at the convolution layer, and the pool size at the pooling layer. Based on previous literature, we have used ReLU as an activation function. The best-performing CNN models have been constructed based on the specifications detailed in Table 10.

**Table 10:** Optimal hyper-parameter values of the fitted CNN models.

Specifications	Original series			Residual series		
	Wheat	Sugarcane	Groundnut	Wheat	Sugarcane	Groundnut
No. of input lags	3	2	3	2	4	3
No. of filters at the convolution layer	6	8	7	6	3	4
Kernel size at the convolution layer	2	2	2	2	2	2
Pool size at the pooling layer	2	1	2	1	3	2
Total no. of parameters	25	33	29	25	13	17

### Fitting of the RNN models

RNNs perform better in predicting sequential, non-linear behavior of the series. Each series has already been duly assessed for the existence of non-linearity. Subsequently, by altering the range of nodes from 1 to 6 at the input layer and 1 to 10 at the hidden layer, the best configuration of hyper-parameters has been acquired. Specifications of the selected RNN models for both original and residual series are provided in Table 11.

**Table 11:** Optimal hyper-parameter values of the fitted RNN models.

Specifications	Original series			Residual series		
	Wheat	Sugarcane	Groundnut	Wheat	Sugarcane	Groundnut
No. of input lags	1	2	4	5	1	1
No. of hidden units	10	5	7	2	6	1
Total no. of parameters	131	41	71	11	55	5

### Fitting of the LSTM models

The structure of the LSTM models studied in this work comprises an input layer, a hidden layer with LSTM cells as hidden nodes, and an output layer with a single output node. Because of its flexibility to apply multiple learning rates for different parameters, adaptive moment estimation (Adam), a prominent variant of the stochastic gradient descent (SGD) method, has been used for loss function optimization^61^. To obtain the optimal set, multiple configurations of the LSTM model have been explored by varying the different hyper-parameters. The LSTM tuning involves considering hyper-parameters such as the number of input and hidden nodes, batch size, and the number of epochs. These hyper-parameters serve not only to govern the model’s architecture and topology, but also to optimize key parameters like biases and weights. Several automated approaches are available in the literature that can potentially be used for hyper-parameter tuning. Out of these techniques, we have used the grid search method, which examines all the possible combinations of hyper-parameters. After trying various combinations, substantial effects have been observed for altering the number of input and hidden nodes, whereas the number of epochs and batch size have shown better results when set to 300 and 1, respectively. The trade-off between the number of (trainable) parameters and the error metrics is also considered for giving due weightage to parsimony. The outcomes of the eventual optimised configuration of the input and hidden nodes are depicted in Table 12.

**Table 12:** Optimal hyper-parameter values of the fitted LSTM models.

Specifications	Original series			Residual series		
	Wheat	Sugarcane	Groundnut	Wheat	Sugarcane	Groundnut
No. of input lags	5	2	4	1	1	6
No. of hidden units	10	7	3	1	8	10
Total no. of parameters	491	260	64	14	329	491

## Discussion

**Figure 3:**
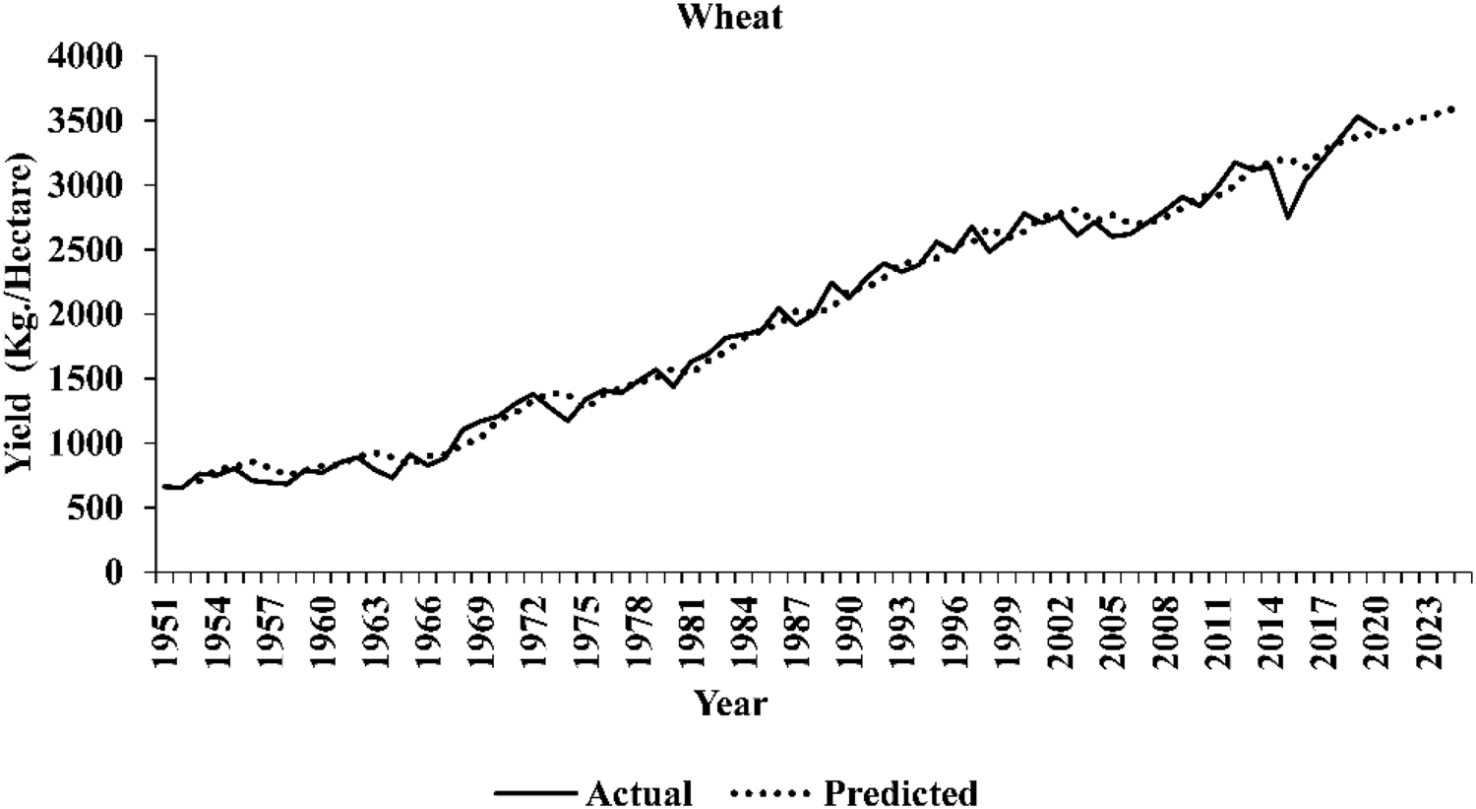
Original series and best-predicted series (by the ARIMAX–LSTM model) for wheat yield in India.

**Figure 4:**
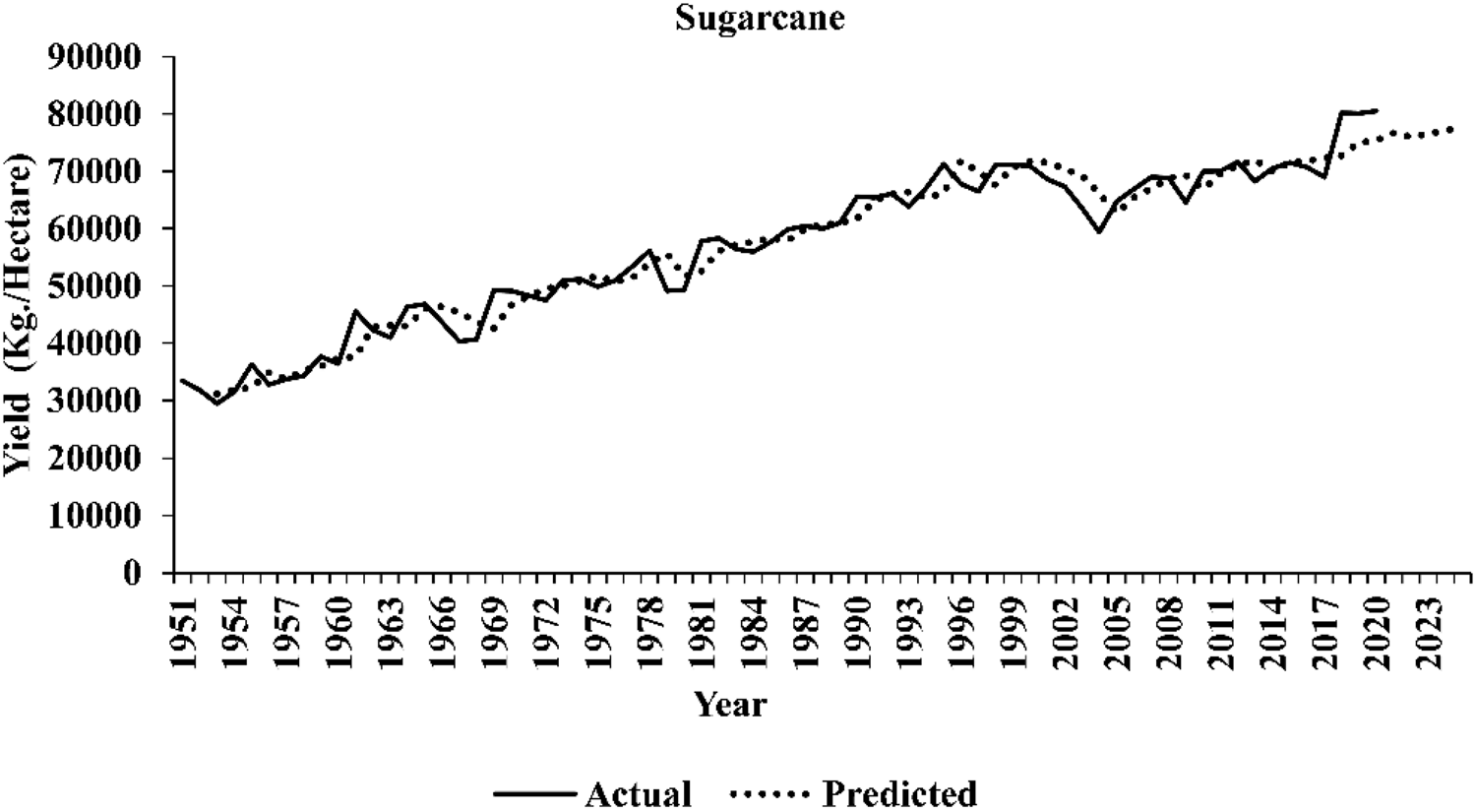
Original series and best-predicted series (by the ARIMAX–LSTM model) for sugarcane yield in India.

**Figure 5:**
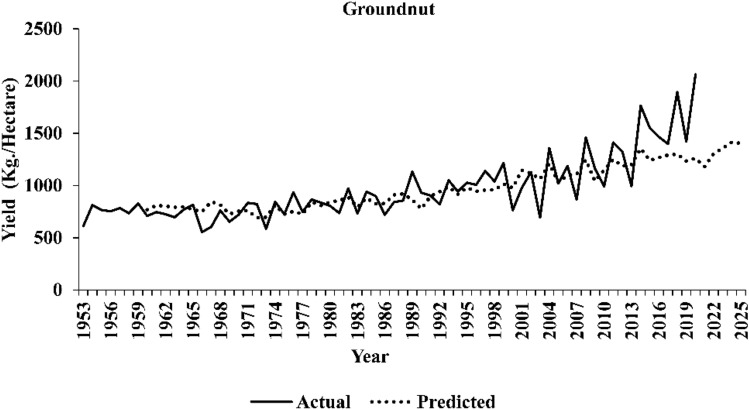
Original series and best-predicted series (by the ARIMAX–LSTM model) for groundnut yield in India.

**Figure 6:**
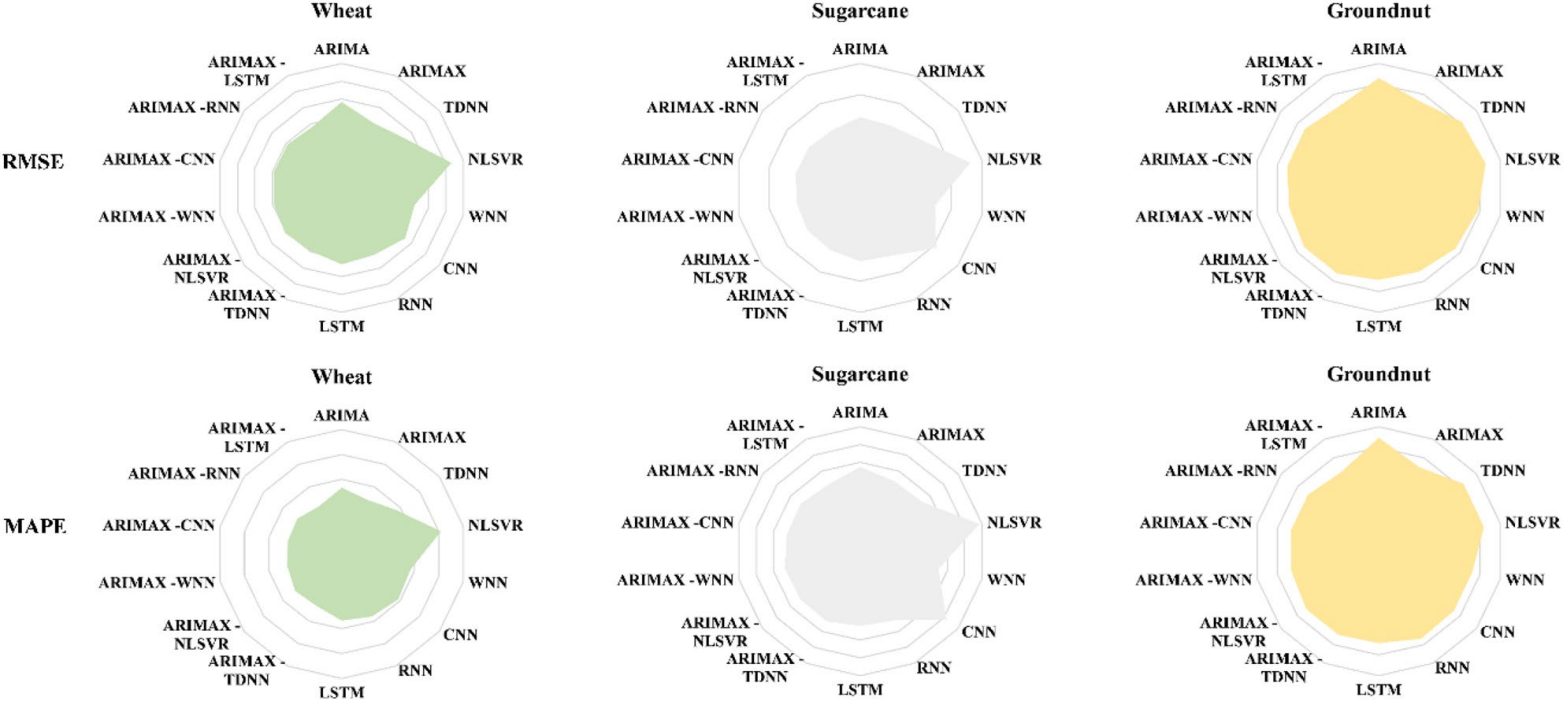
Radar plot of RMSE and MAPE values for the time series models under study.

The comparative assessment of out-of-sample accuracy for different time series models under consideration is given in Table 13. The ARIMAX-LSTM hybrid models appear to have demonstrated superior performance in forecasting yield series, faring better than all the other models. This implies that the forecasted series obtained through the ARIMAX-LSTM framework tends to align more closely with the actual yield series values. The plots of the original series and predicted series by the best performing ARIMAX-LSTM model are shown in Figs. 3, 4, and 5, respectively. The plots clearly show that the ARIMAX-LSTM hybrid models have effectively captured the trends and trajectories of yield movements. Yield forecasts for the next five years (2021–2025) by these models have also been provided in Table 14. Figure 6 displays a radar plot depicting the error metrics (RMSE and MAPE values) for various models under study.

**Table 13:** Forecasting accuracies of the time series models under study.

Model	Wheat		Sugarcane		Groundnut	
	RMSE	MAPE	RMSE	MAPE	RMSE	MAPE
ARIMA	241.79	5.33	4542.02	4.74	529.45	27.34
ARIMAX	203.23	4.82	4444.58	4.37	460.83	22.47
TDNN	224.71	5.65	4952.37	4.44	510.83	26.06
NLSVR	315.85	8.16	7224.47	6.85	526.28	25.94
WNN	210.24	5.66	4922.32	4.49	490.05	23.03
CNN	227.87	5.82	6201.06	6.19	470.88	23.06
RNN	209.64	5.57	4808.26	4.32	447.08	23.39
LSTM	215.31	5.36	4710.83	4.20	444.03	22.19
ARIMAX -TDNN	197.98	4.64	4433.56	4.38	459.13	22.35
ARIMAX -NLSVR	203.36	4.77	4347.60	4.32	459.14	22.36
ARIMAX -WNN	195.39	4.47	4196.79	4.35	441.56	21.55
ARIMAX -CNN	196.37	4.46	4239.21	4.23	453.33	21.76
ARIMAX -RNN	195.04	4.56	4197.64	4.28	454.92	21.87
ARIMAX -LSTM	190.68	4.25	4125.26	4.16	438.58	21.12

**Table 14:** Yield forecasts (Kg./Hectare) by the best performing models for the period of 2021–2025**.**

Year	Best performing model		
	Wheat	Sugarcane	Groundnut
	ARIMAX–LSTM	ARIMAX–LSTM	ARIMAX–LSTM
2021	3436.36	76,677.45	1180.11
2022	3492.55	76,032.79	1306.06
2023	3532.78	76,537.18	1358.41
2024	3573.02	77,153.6	1417.00
2025	3613.26	77,788.84	1401.78

It is also noteworthy to mention that despite the presumption of linearity, meticulous selection of area under irrigation (%) as an auxiliary variable has mediated the outperformance of the ARIMAX model over the univariate non-linear models. As we confront evolving climatic outlooks, the importance of irrigation will become even more pronounced. By reducing reliance on precipitation and maintaining a balanced level of moisture in the soil, irrigated farming can show more resilience to shifts in weather patterns. However, the supremacy of the ARIMAX-LSTM models over the other ARIMAX-based hybrid models is due to the inbuilt ability of the LSTM model to process any sequential data effectively. Its unique structural make up has helped to provide a more comprehensive view of the complex time series data, encompassing both short-term and long-term insights. The competitive advantages of using LSTM in capturing residual patterns were also evidenced in the studies of Manowska et al.^62^ for natural gas consumption forecasting, Wu et al.^63^ for precipitation amount and drought forecasting, Dave et al.^64^ for export forecasting, Khozani et al.^65^ for groundwater level forecasting, etc.

Outcomes emanated from this investigation also suggest the superior performance of all the hybrid models over their individual counterparts. As real-world time series data are subjected to shifts, abrupt changes, and evolving patterns, different forecasting techniques excel in their respective domains^66,67^. Hybrid models can leverage the strengths of various methods to adapt to changing conditions, making them more robust in scenarios where the underlying data-generating process may vary over time. This adaptability is especially valuable for dealing with data from domains where external factors can significantly influence the time series, such as in this case, yield forecasting of *Rabi* crops^68,69^.

In addition, it has been noticed that the ranking of performance among the individual models is distinct from their hybrid counterparts. To illustrate, the performance hierarchy of the non-linear models is as follows: LSTM > RNN > WNN > TDNN > CNN > NLSVR. Conversely, within the hybrid framework, a different performance hierarchy has been observed: LSTM > WNN > CNN > RNN > TDNN > NLSVR. It clearly indicates that the comparative performance of individual models cannot be generalized for the comparison of their hybrid structures, emphasizing the importance of data-driven forecasting exercises.

## Conclusions

In this study, we have employed different ARIMAX-based hybrid models and compared their performances with their individual counterparts as well as with the ARIMA model for yield forecasting of major *Rabi* crops in India. It has been observed that the ARIMAX–LSTM modeling combination has provided better forecasts than other time series models, as evidenced by various accuracy measures. For these models, an average improvement of RMSE and MAPE values has been observed to be 10.41% and 12.28%, respectively over all other competing models and 15.83% and 18.42%, respectively over the benchmark ARIMA model. It can also be inferred that the inclusion of area under irrigation (%) as an exogenous variable in the ARIMAX framework and the inbuilt ability of the LSTM model to process complex non-linear patterns have greatly improved the forecasting accuracy. The performance supremacy of other hybrid models over their individual counterparts has also been evident. It is also suggested to avoid any performance generalization of individual models for their hybrid structures. Future works are expected to explore the performance of other hybrid structures such as ARIMA–NARX, ARIMA–NLSVRX, ARIMA–LSTMX, etc. for agricultural yield forecasting.

## Data Availability

The data that support the findings of this study are available on request from the first author: Pramit Pandit.
